# Associations between night work and BMI, alcohol, smoking, caffeine and exercise - a cross-sectional study

**DOI:** 10.1186/s12889-015-2470-2

**Published:** 2015-11-12

**Authors:** Hogne Vikanes Buchvold, Ståle Pallesen, Nicolas M. F. Øyane, Bjørn Bjorvatn

**Affiliations:** Department of Global Public Health and Primary Care, University of Bergen, Bergen, Norway; Norwegian Competence Center for Sleep Disorders, Haukeland University Hospital, Bergen, Norway; Department of Psychosocial Science, University of Bergen, Bergen, Norway

**Keywords:** Shift work, Night work, BMI, Obesity, Alcohol, Caffeine, Smoking, Exercise

## Abstract

**Background:**

Shift work is associated with negative health effects. Increased prevalence of several cardiovascular risk factors among shift workers/night workers compared with day workers have been shown resulting in increased risk of cardiovascular events among shift workers and night workers. Previous studies have taken a dichotomous approach to the comparison between day and night workers. The present study uses a continuous approach and provides such a new perspective to the negative effects of night work load as a possible risk factor for undesirable health effects.

**Methods:**

This cross sectional study (The SUrvey of Shift work, Sleep and Health (SUSSH)) uses data collected from December 2008 to March 2009. The study population consists of Norwegian nurses. The study collected information about demographic and lifestyle factors: Body Mass Index (BMI), smoking habits, alcohol consumption, caffeine consumption and exercise habits. The lifestyle parameters were evaluated using multiple hierarchical regression and binary logistic regression. Number of night shifts worked last year (NNL) was used as operationalization of night work load. Adjustment for possible confounders were made. Obesity was defined as BMI > 30. Alcohol Consumption was evaluated using the short form of the Alcohol Use Disorders Identification Test Consumption (AUDIT-C). Data were analyzed using SPSS version 22.

**Results:**

We had data from 2059 nurses. NNL was significantly and positively associated with BMI, both when evaluated against BMI as a continuous parameter (*Beta* = .055, p < .05), and against obesity (OR = 1.01, 95 % CI = 1.00-1.01). The AUDIT-C score was significantly and positively associated with hours worked per week (OR = 1.03, 95 % CI = 1.01-1.05).

**Conclusions:**

We found a positive significant association between night work load and BMI. This suggests that workers with a heavy night work load might need special attention and frequent health checks due to higher risk of undesirable health effects.

## Background

Shift work is consistently shown to be associated with adverse health effects, i.e. gastrointestinal complaints, sleep difficulties, cancer, cardiovascular and metabolic diseases, and mental health problems [1–4]. In industrialized countries many sectors rely on 24 hours services, for instance the health care sector. With increasing evidence in terms of adverse effects on health, more attention and research has been directed to this field. In particular, much emphasis has been put on the possible increased risk of cardiovascular disease among shift workers, although a causal relationship remains unclear [5–8].

Cardiovascular disease is one of the leading causes of death in industrialized countries. Over the years several cardiovascular risk factors for have been identified, and much effort has been devoted in order to reduce or eliminate the impact from these. Especially among sub-populations/high risk groups, risk stratification and primary prevention has been advocated, such as smoking cessation, increased physical activity, moderate alcohol intake, and weight management [9].

Several studies have focused on relative differences in prevalence of cardiovascular risk factors of shift workers/night workers compared to day workers. Shift and night workers have higher prevalence of risk factors such as smoking, dyslipidemia, and weight gain when comparing to day workers [5, 6]. Biggi et al. found that the cluster of independent risk factors collectively termed metabolic syndrome was increased among night workers compared to day workers [7]. Bøggild et al. concluded in a metaanalysis that shift work represented a 40 % increase in the risk of cardiovascular disease [8].

As a result of the increasing evidence supporting negative health effects of shift work, newly published studies suggest that countermeasures are needed to reverse this. Different measures for primary prevention have been proposed to counteract the negative effects of shift work: for instance, proper work scheduling, exercise, and dietary guidelines [10, 11]. In addition to longitudinal studies addressing possible causal relationship between shift work and cardiovascular disease, more studies are needed to investigate possible sub-populations among shift workers who have an elevated risk for developing cardiovascular disease.

Most previous studies have taken a dichotomous approach to the comparison of night and day workers in terms of possible cardiovascular risk factors such as weight gain and elevated BMI. The present study instead evaluates the night shift work load effect on BMI, alcohol consumption, smoking habits, caffeine consumption, and exercise habits using the number of night shifts worked the last year as a predictor. Our design may help to investigate further whether workers with a heavy night shift work load might need more frequent health checks or more direct countermeasures due to increased risk of undesirable health effects and habits.

## Methods

### Design

The data were stemmed from “The SUrvey of Shift work, Sleep and Health” (SUSSH). This cross-sectional study was conducted from December 2008 to March 2009. The population consisted of registered members of the Norwegian Nurses Organization (NNO), which include most of the nurses working in Norway today. In January 2009 there were 87083 registered members of NNO. A stratified sample (*N* = 6000) comprising a total of five strata; each containing 1200 nurses holding at least a 50 % work position, was randomly selected from the member registry of the NNO. The criteria for the different strata were time elapsed since graduation: less than 12 months (stratum 1), 1–3 years (stratum 2), >3-6 years (stratum 3), >6-9 years (stratum 4) and >9-12 years (stratum 5). Each nurse in the sample received a questionnaire by postal mail. After completing the questionnaire, the respondents could return them in a pre-paid envelope. Two reminders were sent to those who did not respond. An internet version of the questionnaire was available for those who preferred to complete the questionnaire online. A total of 600 letters were returned due to wrong addresses. As a result the final sample consisted of 5400 nurses, of which 2059 participated in the survey, yielding a response rate of 38.1 %.

### Data

The questionnaire covered demographic factors in terms of sex and age, marital status, and whether the responders had children living at home. Responders were also asked for their working schedule: day only, evening only, day and evening, three shift rotation, night only, or another schedule including night work. The questionnaire also covered how long they had been working this schedule, and how long they had worked as a nurse. The nurses were asked to indicate the number of night shifts they had worked the last year (NNL). Furthermore they were asked to report average work hours per week, and their percentage of a full time equivalent work position (50-75 %, 76-90 % and above 90 %).

### BMI

Body Mass Index was calculated conventionally using weight over the square of height in meters. The nurses self-reported height and weight in the questionnaire. We had data on weight and height for a total of 2038 nurses. Obesity was defined as BMI > 30.

### Smoking habits

The nurses were asked if they smoked daily (yes/no). Those who smoked were further asked to provide number of cigarettes smoked daily. In our cohort 214 nurses were daily smokers. Number of cigarettes smoked daily comprised the dependent variable in the linear regression analysis wheras daily smoking (yes/no) was used as dependent variable in a logistic regression model.

### Alcohol consumption

Alcohol Consumption was evaluated using the short form of the Alcohol Use Disorders Identification Test Consumption (AUDIT-C). AUDIT-C is a self report instrument with three items measuring alcohol consumption. The instrument appears to be a practical, valid primary screening test for heavy drinking and/or active alcohol abuse or dependence [12]. A score of 3 or higher points on the AUDIT-C might indicate potential alcohol misuse. In a primary care setting a threshold score of 3 or higher in females, and 4 or higher in males simultaneous maximized sensitivity and specificity [13]. We had data for 2021 nurses. In our analysis we used the composite AUDIT-C score as a parameter for potential alcohol misuse in a hierarchical regression analysis, and the dichotomous AUDIT C score (cut off: ≥3 for females and ≥4 for males) as dependent variables in logistic regression analyses. The Cronbach’s alpha for AUDIT C was 0.68 in the present study.

### Caffeine consumption

Nurses were asked to indicate average number of caffeine containing units consumed per day. The questionnaire did not differentiate between drinks with different total caffeine content. For example, one unit would be one cup of coffee or a glass of coca cola. 2050 nurses responded to this question. Caffeine consumption was evaluated as a dichotomous parameter (drinking 3 or more caffeine containing units vs. less than 3 units per day).

### Exercise habits

Exercise was measured by an item asking for hours of sweaty exercise per week (0, <1 h, 1-2 h, ≥3 hours), and was answered by 1971 nurses. We collapsed exercise data into two groups (<1 h and ≥1 h per week). In a large female cohort study at least one hour walking per week predicted lower risk for cardiovascular disease [14].

### Statistical analyses

SPSS version 22 was used for the analyses. In the linear multiple hierarchical regression models we wanted to investigate what kind of effect number of nights worked the last year (NNL) had on: BMI, alcohol consumption, smoking habits, when adjusting for possible confounding factors. Caffeine consumption was excluded from the multiple hierarchical regression model due to violation of normality assumption. Each of the lifestyle parameters were analyzed separately, using the same type of multiple hierarchical regression model. Step 1 included demographic factors: sex and age. Step 2 included hours worked per week and the duration of experience with night work (more or less than five years). In step 3. children living at home, and NNL were included in the model.

Furthermore, binary logistic regression analyses were used to investigate whether NNL was significantly related to the dichotomized (based on cut-offs) lifestyle parameters. Both crude and adjusted analyses were undertaken for all dependent parameters. Caffeine consumption and exercise habits were included as dependent variables in these models in addition to obesity, AUDIT-C, and smoking. The adjusted logistic regression models controlled for the same possible confounders as in the linear multiple hierarchical regression models described above. There is variation in the number of participants in the different models due to missing data, as indicated in the tables and data section. In the adjusted analyses, n will naturally be lower, since only participants who have answered all questions in the model will be included in the analysis.

### Ethics

The Regional Committee for Medical and Health Research Ethics of Western Norway (REK-West) approved the study.

## Results

### Demographics

The mean age was 33.1 years (SD 8.2), range 21 to 63 years. The study population consisted predominately of females (90.6 %). The nurses worked on average 33.9 hours per week (SD 6.5), 55.8 % of the nurses reported holding a working position that exceeded 90 %. They had worked on average 5.2 years as nurses (SD 4.3). In all 76.0 % worked in medical-surgical hospital departments, 13.8 % worked in psychiatric departments, 3.6 % in nursing homes, and 3.7 % worked in home care services, and 2.9 % in others respectively. Three shift rotation was most common (56.2 %), followed by two shift (24.8 %), night shift only (8.2 %), day shift only (7.5 %), other schedules with day and night 2.8 %, and evening shift only 0.2 %. For number of nights worked the last year a mean of 25.0 (SD 28.9) night shifts, with range from 0 to 206 were reported. A total of 66.1 % had schedules which included night work for less than five years, 33.9 % for more than five years. Sweaty exercise for ≥1 hour per week on average was reported by 28.2 % of the nurses reported. Demographic characteristics are shown in Table 1.

### BMI

Mean BMI was 24.4 (SD 4.0). Using BMI as the dependent variable in the hierarchical regression analysis we found that step 1 (age and sex) explained 4.5 % of the variance (Table 2). Step 2 and 3 did not explain significant proportions of the variance. After step 3 the model as whole explained 4.9 % of the variance F(6,1668) = 14.18, *p* < .05. Evaluating each of the independent variables separately, number of nights worked the last year (NNL) was statistically significant and positively related to BMI (*β* = .055, *p* < .05). NNL was also significant and positively associated to BMI when adding exercise as an independent predictor to the same model ((*β* = .057, *p* < 0.05). Data not shown. Age was positively related to BMI (*β* = .145, *p* < 0.05). Sex was positively and significantly related to BMI: females had lower BMI than males (*β* = −.147 *p* < 0.05). In our logistic regression model, NNL was positively associated (OR = 1.01, 95 % CI = 1.00-1.01) with obesity (BMI > 30) (Table 3).

**Table 1:** Demographic characteristics of nurses with different kinds of night work load

	Number of nights worked last year		
	0 (*n* = 568)	1-30 (*n* = 860)	>30 (*n* = 631)
Age (*n* = 2057)	34.9 (34.2-35.6)	32.4 (31.8-32.9)	32.4 (31.8-33.0)
Gender (% female) (*n* = 2049)	92.6 % (90.4-94.7)	92.5 % (90.7-94.3)	86.3 % (83.6-89.0)
Hours Worked Per Week (*n* = 2017)	34.0 (33.4-34.6)	33.7 (33.2-34.1)	34.2 (33.6-34.7)
Average hours Sleep per Night (*n* = 2048)	6.9 (6.8-7.0)	6.9 (6.8-7.0)	7.0 (6.9-7.0)
Worked Schedule including Night Work (% > 5 years) (*n* = 1750)	26.7 % (21.8-31.5)	29.5 % (26.4-32.7)	43.5 % (39.6-47.5)
Sweaty Excercise (% > 1 hour per week) (*n* = 1971)	64.1 %(60.0-68.1)	67.4 % (64.2-70.1)	65.7 % (61.2-69.5)
BMI (*n* = 2038)	24.4 (24.0-24.7)	24.1 (23.9-24.4)	24.8 (24.4-25.1)
Obesity (*n* = 2038)	10.0 % (7.6-12.6)	6.6 % (4.9-8.3)	11.7 % (9.2-14.3)
AUDIT-C (*n* = 2021)	3.3 (3.2-3.5)	3.7 (3.6-3.8)	3.8 (3.7-4.0)
Caffeine Containing Drinks per Day (*n* = 2050)	3.2 (3.0-3.5)	2.8 (2.7-3.0)	3.2 (3.0-3.4)
Daily Smokers (*n* = 1956)	12.9 % (10.1-15.7)	9.7 % (7.7-11.7)	10.7 % (8.8-13.2)
Cigarettes per day among daily smokers (*n* = 214)	10.0 (8.6-11.4)	9.0 (7.9-10.1)	9.1 (7.9-10.3)

Confidence Intervals 95 % in parenthesis. *BMI* body mass index. Obesity defined as BMI >30. AUDIT-C = The *AUDIT* alcohol consumption questions

**Table 2:** Multiple hierarchical regression analyses with BMI, AUDIT-C, and number of cigarettes daily as dependent variable

	BMI (*n* = 1674)		AUDIT-C (*n* = 1622)		Number of cigarettes smoked daily (*n* = 184)	
	*β*	*ΔR* ^*2*^	*β*	*ΔR* ^*2*^	*β*	*ΔR* ^*2*^
Step 1		0.045*		0.063*		0.113*
Age	0.140*		−0.203*		0.288*	
Gender (1 = females)	−0.153*		−0.158*		−0.157*	
Step 2		0.0002		0.018*		0.016
Age	0.137*		−0.179*		0.246*	
Gender	−0.154*		−0.141*		−0.152*	
Average hours worked per week	−0.009		0.120*		0.002	
Schedule with Night Work >5 years	0.009		−0.067*		0.134	
Step 3		0.003		0.047*		0.010
Age	0.145*		−0.113*		0.267*	
Gender	−0.147*		−0.132*		−0.157*	
Average hours worked per week	−0.003		0.075*		−0.020	
Schedule with Night Work >5 years	0.001		−0.052*		0.152*	
Children living at Home no/yes	−0.009		−0.229*		−0.097	
NNL	0.055*		0.032		- 0.057	

* Significant finding *p* < .05. *β* Beta coefficient, *ΔR2* R square change, *BMI* body mass index, *AUDIT C* the *AUDIT* alcohol consumption questions, *NNL* number of night shifts worked last year

**Table 3:** Logistic regression analyses predicting effects on lifestyle variables

Independent Variable	Obesity (BMI > 30)				AUDIT-C (Score ≥3 female ≥4 males)				Smoking (yes/no)				Caffeine Consumption (Score ≥ 3)				Exercise Habits (≥1 hour per week)			
	Crude(*n* = 1730-2038)		Adjusted (*n* = 1622)		Crude (*n* = 1711-2011)		Adjusted (*n* = 1612)		Crude (*n* = 1658-1956)		Adjusted (*n* = 1557)		Crude (*n* = 1741-2050)		Adjusted (*n* = 1631)		Crude (*n* = 1678-1971)		Adjusted (*n* = 1575)	
	OR	95 % CI	OR	95 % CI	OR	95 % CI	OR	95 % CI	OR	95 % CI	OR	95 % CI	OR	95 % CI	OR	95 % CI	OR	95 % CI	OR	95 % CI
Age	1.02* n = 2036	1.00-1.04	1.02	0.99-1.04	0.95* n = 2010	0.94-0.97	0.97*	0.95-0.98	0.97* n = 1955	0.95-0.99	0.96*	0.94-0.97	1.10* n = 2048	1.08-1.11	1.10*	1.09-1.14	1.00 n = 1969	0.99-1.01	1.01	0.99-1.02
Gender																				
Female	1.00 *n* = 1836		1.00		1.00 *n* = 1821		1.00		1.00 *n* = 1737		1.00		1.00 *n* = 1849		1.00		1.00 n = 1774		1.00	
Male	1.83* *n* = 192	1.18-2.82	1.45	0.88-2.47	0.82 *n* = 190	0.60-1.13	0.78	0.53-1.15	1.15 *n* = 210	0.69-1.91	1.05	0.59-1.85	3.25* *n* = 191	2.30-4.58	3.17*	2.10-4.81	1.83* *n* = 187	1.34-2.50	1.65*	1.15-2.36
Average hours worked /week	1.00 *n* = 1996	0.98-1.02	0.99	0.97-1.02	1.03* n = 1971	1.02-1.05	1.03*	1.01-1.05	0.99 *n* = 1915	0.97-1.02	1.00	0.98-1.03	1.02* *n* = 2008	1.01-1.03	1.01	1.00-1.03	1.02* *n* = 1932	1.01-1.04	1.01	0.99-1.03
Schedule with night work																				
<5 years	1.00 *n* = 1141		1.00		1.00 *n* = 1130		1.00		1.00 *n* = 1097		1.00		1.00 *n* = 1150		1.00		1.00 *n* = 1115		1.00	
>5 years	1.31 *n* = 589	0.94-1.82	1.07	0.74-1.54	0.59* n = 581	0.47-0.73	0.75*	0.59-0.95	0.92 *n* = 561	0.66-1.28	1.15	0.80-1.65	1.70* *n* = 591	1.39-2.08	0.95	0.75-1.21	1.03 *n* = 563	0.83-1.28	1.10	0.86-1.41
Children living at Home																				
No	1.00 *n* = 982		1.00		1.00 *n* = 971		1.00		1.00 *n* = 941		1.00		1.00 *n* = 989		1.00		1.00 *n* = 944		1.00	
Yes	1.11 *n* = 966	0.82-1.51	1.08	0.75-1.54	0.48* *n* = 953	0.40-0.59	0.60*	0.47-0.76	1.05 *n* = 932	0.78-1.40	1.12	0.84-1.68	0.75* *n* = 969	0.63-0.90	0.83	0.65-1.00	0.61* *n* = 941	0.50-0.75	0.59*	0.47-0.75
NNL	1.01* n = 2038	1.00-1.01	1.01*	1.00-1.01	1.00 n = 2011	1.00-1.01	1.00	1.00-1.01	1.00 n = 1956	1.00-1.01	1.00	0.99-1.00	1.00* n = 2050	1.00-1.01	1.00*	1.00-1.01	1.00 n = 1971	1.00-1.01	1.00	1.00-1.00

* Significant finding, *p* < .05. *NNL* number of night shifts worked last year

### Alcohol consumption

Mean score for AUDIT-C was 3.7 (SD 2.0). Using the same hierarchical regression model, we found that after step 3 the model as a whole explained 12.5 % of the variance F(6,1668) = 40.8 *p* < .05 (Table 2). Age was negatively associated with alcohol consumption (*β* = −.113, *p* < .05). Females reported a significantly lower alcohol consumption than males (*β* = −.132, *p* < .05). Hours worked per week was significant and positively associated to alcohol consumption (*β* = .075, *p* < .05). Those who have had worked schedules including night work for over 5 years had lower consumption (*β* = −.052 *p* < .05) than those with less night work experience. Those who had children living at home had a lower AUDIT-C score compared to nurses without children living at home (*β* = −.229 *p* < .05). NNL was not significantly related to the AUDIT-C composite score (*β* = .032 *p* = .167). In our logistic regression model we found no significant association with NNL, however there was a significant positive relationship (OR = 1.03, 95 % CI = 1.01-1.05) between average working hours per week and the AUDIT-C score. Children living at home were inversely related to the AUDIT C score (OR = 0.60, 95 % CI = 0.47-0.76). Age was inversely related to the AUDIT-C score (OR = 0.97 CI = 0.95-0.98). Those who have had worked schedules including night work for over 5 years had significantly lower AUDIT-C score (OR = 0.75 CI = 0.59-0.95) (Table 3).

### Smoking

Mean cigarettes smoked daily were 9.4 (SD 5.2) among daily smokers. Using the same hierarchical regression model, we found that after step 3 the model as a whole explained 13.9 % of the variance F(6,178) = 4.8 *p* < .05 (Table 2). Age was positively associated with cigarettes smoked (*β* = .267 *p* < 0.5). Males were smoking more than females (*β* = .-157 *p* < .05). Those who have had worked schedules including night work for over 5 years smoked significantly more (*β* = .152 p < 0.5). Evaluating smoking using logistic regression we did not find any significant associations except age (OR = 0.96, 95 % CI = 0.94-0.97) (Table 3).

### Caffeine consumption

Mean caffeine containing drinks per day were 3.0 (SD 2.7). Evaluating caffeine consumption using logistic regression we found age (OR = 1.10, 95 % CI = 1.09-1.14), male gender (OR = 3.17, 95 % CI = 2.10-4.81) and NNL (OR = 1.00, 95 % CI = 1.00-1.01) to all be significantly and positively associated with caffeine consumption (≥3 drinks per day).

### Exercise habits

We found males to be exercising significantly more (OR = 1.65 95 % CI = 1.15-2.36) than females, and that those with children living at home exercised significantly less (OR = 0.59 95 % CI = 0.47-0.75) than those without children living at home. NNL was not associated with exercise habits.

## Discussion

Our findings suggest that there is a positive association between night work load and BMI, even when controlling for several relevant confounders. The association was significant both when using BMI as a continuous parameter, and when evaluated as obesity (BMI >30). Ramin et al. found that higher levels of average night shifts per month in American nurses were significantly associated with increased risk of obesity [15]. This is consistent with our finding. Other studies have taken a dichotomous approach and shown a significant difference in BMI or weight gain between night and day workers. For instance, Biggi et al. found that night workers had significantly higher BMI than day workers [7]. Metabolic syndrome is being defined as a cluster of cardiovascular risk factors: obesity, dyslipidemia, hypertension, and impaired glucose tolerance. Bacquer et al. found in a longitudinal study that rotating shift work increased the risk of metabolic syndrome. The risk was graded with respect to the number of years with shift work [16]. Furthermore several studies have looked directly at cardiovascular risk and found that shift workers are at higher risk [8, 17]. There is, however, some controversy regarding increased incidence among shift workers for ischemic heart disease [18].

Due to the cross sectional design of the current study, no causual relationship can be established. Still, some notions about possible processes involved seem warranted. One possible underlying mechanism explaining our results is disruption of the circadian rhythm, which may impair glucse metabolism and lipid homeostasis [19]. Another possible explanation is irregular sleep-wake cycle or short sleep duration which is associated with heavy night work load. Bjorvatn et al. have previously reported a clear association between short sleep duration and elevated BMI and obesity [20]. Short sleep duration has been shown to influence hormones related to appetite regulation [19]. Altered eating behavior is another proposed mechanism; Wong et al. found that among nurses shift work was positively associated with abnormal eating behavior [21]. Studies indicate that the total energy intake in night and day workers does not differ significantly, but the quality of diet and distribution of energy intake might explain the observed differences [11]. In summary, both biological and behavioral mechanisms are believed to contribute to the increased BMI observed among those with altered sleep-wake cycle [11, 22].

We did not find any significant association between alcohol consumption and night work load. Hermanson et al. examined 990 subjects working day, two-shift or three shifts schedules with AUDIT and biochemical parameters indicating potential misuse: carbohydrate-deficient transferrin (CDT) and Gamma-glutamyl transferase (GGT). Using these three parameters they did not find a higher level of risky alcohol consumption among shift workers compared to day workers: however, two shift-workers appeared to drink less [23]. Ohida et al. found that among Japanese female nurses there was an positive association between working night shift and using alcohol as sleep aid [24]. We found that hours worked per week were positively correlated with alcohol consumption, suggesting that high work load might lead to higher alcohol consumption, which is consistent findings from a large systematic review by Virtanen et al. [25]. Wong et al. did not find a significant difference in alcohol consumption between ever and never night workers in a large female cohort [26].

We did not find any significant association between night work load and smoking. However, many studies repeat elevated levels of smoking among shift workers [7, 27]. In a large cohort of nurses, Ramin et al. found that night workers were more likely to smoke and consumed more caffeine than those who had never worked night shift [15]. We found a significant positive association between NNL and daily caffeine consumption, which might suggest that caffeine is being used as a stimulant during night work. Shy et al. report that 89 % of emergency residents consumed caffeine during night shifts, with 52 % using it every shift [28].

We did not find any association between night work load and exercise habits. Our dichotomized parameter for exercise habits might not be sensitive enough to unveil any association. An association could theoretically go both ways. A positive association might be explained by increased lifestyle awareness to counteract known negative health effects of night work. A negative association might be due to social and practical constraints and disruption of daily rhythm. Schneider et al. did not find an association between leisure time physical activity and shift work when adjusting for possible confounders [29].

The strengths of the present study are its homogeneous population and size. All variables except caffeine and exercise habits were evaluated using both multiple linear hierarchical regression and binary logistic regression. To further evaluate the possible association between night work load and BMI more studies with prospective designs are warranted [30]. The limitations regarding this study concern its cross-sectional design, and uncertainties regarding data based on subjective reports. The low response rate in this study is unfortunately a part of an increasing problem in epidemiological research. A review by Baruch et al. suggested that most study populations have a response rate around 53 % ± 20 % (1 standard deviation from the mean response rate in this review) [31]. Our response rate (38,1 %) is within this range. Unfortunately, we have no information about the non-responders, making comparative analysis not possible. The low response rate and other methodological issues warrants caution in interpreting results, and exemplifies the need for prospective studies in this research area. There will be some uncertainties regarding nurses own estimation of number of nights worked the last year (NNL). However, many nurses have regular schedules that should make them able to make good estimates of NNL. A potential problem with our study is the “healthy worker effect”: i.e. only those with a tolerance for night work tend to stay in this type of work. Hence, this might have led to an underestimation of the true negative effects of night work.

## Conclusions

This study adds to the growing evidence of the effect of night work on BMI. We also found a consistently higher AUDIT-C score across our models for those with a higher work load which might contribute further to undesirable health effects. Some sub-populations among shift workers, for example those with a heavy night work load, certain chronotypes, and those with high cardiovascular risk at baseline might need special attention and frequent health checks up due to higher risk of undesirable health effects. Earlier introduction of possible counter-measures; for example exercise or, if possible, changes in shift schedule may be needed for those vulnerable to the negative impact of night work.

## References

[1] Knutsson A (2003). Health disorders of shift workers. Occup Med Oxf Engl.

[2] Wise J (2009). Danish night shift workers with breast cancer awarded compensation. BMJ.

[3] Straif K, Baan R, Grosse Y, Secretan B, Ghissassi FE, Bouvard V (2007). Carcinogenicity of shift-work, painting, and fire-fighting. Lancet Oncol.

[4] Marquié J-C, Tucker P, Folkard S, Gentil C, Ansiau D: Chronic effects of shift work on cognition: findings from the VISAT longitudinal study. Occup Environ Med 2014:oemed–2013–101993. doi:10.1136/oemed-2013-101993 10.1136/oemed-2013-101993 25367246

[5] Morikawa Y, Nakagawa H, Miura K, Soyama Y, Ishizaki M, Kido T (2007). Effect of shift work on body mass index and metabolic parameters. Scand J Work Environ Health.

[6] Pietroiusti A, Neri A, Somma G, Coppeta L, Iavicoli I, Bergamaschi A (2010). Incidence of metabolic syndrome among night-shift healthcare workers. Occup Environ Med.

[7] Biggi N, Consonni D, Galluzzo V, Sogliani M, Costa G (2008). Metabolic syndrome in permanent night workers. Chronobiol Int.

[8] Bøggild H, Knutsson A (1999). Shift work, risk factors and cardiovascular disease. Scand J Work Environ Health.

[9] Pearson TA, Blair SN, Daniels SR, Eckel RH, Fair JM, Fortmann SP (2002). AHA guidelines for primary prevention of cardiovascular disease and Stroke: 2002 update: consensus panel guide to comprehensive risk reduction for adult patients without coronary or other atherosclerotic vascular diseases. Circulation.

[10] Pallesen S, Bjorvatn B, Magerøy N, Saksvik IB, Waage S, Moen BE (2010). Measures to counteract the negative effects of night work. Scand J Work Environ Health.

[11] Lowden A, Moreno C, Holmbäck U, Lennernäs M, Tucker P (2010). Eating and shift work - effects on habits, metabolism and performance. Scand J Work Environ Health.

[12] Bush K, Kivlahan DR, McDonell MB, Fihn SD, Bradley KA (1998). The AUDIT alcohol consumption questions (AUDIT-C): an effective brief screening test for problem drinking. Ambulatory Care Quality Improvement Project (ACQUIP). Alcohol use disorders identification test. Arch Intern Med.

[13] Bradley KA, DeBenedetti AF, Volk RJ, Williams EC, Frank D, Kivlahan DR (2007). AUDIT-C as a brief screen for alcohol misuse in primary care. Alcohol Clin Exp Res.

[14] Lee IM, Rexrode KM, Cook NR, Manson JE, Buring JE (2001). Physical activity and coronary heart disease in women: is “no pain, no gain” passé?. JAMA.

[15] Ramin C, Devore EE, Wang W, Pierre-Paul J, Wegrzyn LR, Schernhammer ES: Night shift work at specific age ranges and chronic disease risk factors. Occup Environ Med 2014:oemed–2014–102292. doi: 10.1186/2052-4374-26-6 10.1136/oemed-2014-102292 PMC4289641 25261528

[16] De Bacquer D, Van Risseghem M, Clays E, Kittel F, De Backer G, Braeckman L (2009). Rotating shift work and the metabolic syndrome: a prospective study. Int J Epidemiol.

[17] Ellingsen T, Bener A, Gehani AA (2007). Study of shift work and risk of coronary events. J R Soc Promot Health.

[18] Frost P, Kolstad HA, Bonde JP (2009). Shift work and the risk of ischemic heart disease - a systematic review of the epidemiologic evidence. Scand J Work Environ Health.

[19] Kim TW, Jeong J-H, Hong S-C (2015). The Impact of Sleep and Circadian Disturbance on Hormones and Metabolism. Int J Endocrinol.

[20] Bjorvatn B, Sagen IM, Øyane N, Waage S, Fetveit A, Pallesen S (2007). The association between sleep duration, body mass index and metabolic measures in the Hordaland Health Study. J Sleep Res.

[21] Wong H, Wong MCS, Wong SYS, Lee A (2010). The association between shift duty and abnormal eating behavior among nurses working in a major hospital: a cross-sectional study. Int J Nurs Stud.

[22] Bayon V, Leger D, Gomez-Merino D, Vecchierini M-F, Chennaoui M (2014). Sleep debt and obesity. Ann Med.

[23] Hermansson U, Knutsson A, Brandt L, Huss A, Rönnberg S, Helander A (2003). Screening for high-risk and elevated alcohol consumption in day and shift workers by use of the AUDIT and CDT. Occup Med Oxf Engl.

[24] Ohida T, Kamal A, Sone T, Ishii T, Uchiyama M, Minowa M (2001). Night-shift work related problems in young female nurses. J Occup Health.

[25] Virtanen M, Jokela M, Nyberg ST, Madsen IEH, Lallukka T, Ahola K (2015). Long working hours and alcohol use: systematic review and meta-analysis of published studies and unpublished individual participant data. BMJ.

[26] Wang X-S, Travis RC, Reeves G, Green J, Allen NE, Key TJ (2012). Characteristics of the million women study participants who have and have not worked at night. Scand J Work Environ Health.

[27] Puttonen S, Härmä M, Hublin C (2010). Shift work and cardiovascular disease – pathways from circadian stress to morbidity. Scand J Work Environ Health.

[28] Shy BD, Portelli I, Nelson LS (2011). Emergency medicine residents’ use of psychostimulants and sedatives to aid in shift work. Am J Emerg Med.

[29] Schneider S, Becker S (2005). Prevalence of physical activity among the working population and correlation with work-related factors: results from the first German National health survey. J Occup Health.

[30] van Drongelen A, Boot CRL, Merkus SL, Smid T, van der Beek AJ (2011). The effects of shift work on body weight change - a systematic review of longitudinal studies. Scand J Work Environ Health.

[31] Baruch Y, Holtom BC (2008). Survey response rate levels and trends in organizational research. Hum Relat.

